# Strategies for mitigating radiation damage and improving data completeness in 3D electron diffraction of protein crystals

**DOI:** 10.1107/S2059798325011258

**Published:** 2026-01-01

**Authors:** Alaa Shaikhqasem, Farzad Hamdi, Lisa Machner, Christoph Parthier, Constanze Breithaupt, Fotis L. Kyrilis, Stephan M. Feller, Panagiotis L. Kastritis, Milton T. Stubbs

**Affiliations:** a Institut für Biochemie und Biotechnologie Martin-Luther-Universität Halle-Wittenberg Halle (Saale) Germany; b Charles-Tanford-Proteinzentrum Martin-Luther-Universität Halle-Wittenberg Halle (Saale) Germany; c ZIK HALOmem, Biozentrum Martin-Luther-Universität Halle-Wittenberg Halle (Saale) Germany; d Institute of Molecular Medicine Martin-Luther-Universität Halle-Wittenberg Halle (Saale) Germany; e Institute of Chemical Biology National Hellenic Research Foundation 11635Athens Greece; f Mitteldeutsches Zentrum für Struktur und Dynamik der Proteine Martin-Luther-Universität Halle-Wittenberg Halle (Saale) Germany; University of Manchester, United Kingdom

**Keywords:** electron diffraction, radiation damage, data completeness, 3D-ED, MicroED

## Abstract

While 3D electron diffraction (3D-ED or microcrystal electron diffraction; MicroED) has emerged as a promising method for protein structure determination, its applicability is hindered by a high susceptibility to radiation damage, leading to a decreasing signal-to-noise ratio in consecutive diffraction patterns that limits the quality (resolution and redundancy) of the data. In addition, data completeness may be restricted due to the geometrical limitations of current sample holders and stages. Although specialized equipment can overcome these challenges, many laboratories do not have access to such instrumentation. In this work, we introduce an approach that addresses these issues using a commonly available 200 keV cryo-electron microscope. The multi-position acquisition technique that we present here combines (i) multiple data acquisitions from a single crystal over several tilt ranges and (ii) merging data from a small number of crystals each tilted about a different axis. The robustness of this approach is demonstrated by the *de novo* elucidation of a protein–peptide complex structure from only two orthorhombic microcrystals.

## Abstract 2

This work demonstrates that multi-position data acquisition mitigates radiation damage in electron diffraction of three-dimensional protein crystals and that merging data from selected crystals in distinct orientations enhances completeness. Application of the two approaches enables high-quality structural models to be obtained from limited or sensitive samples.

## 1. Introduction

X-ray crystallography has represented a major workhorse for biological macromolecular structure determination for over six decades. The low X-ray scattering cross section of light atoms typically requires the use of macroscopic (≳10^−4^ m) crystals to achieve sufficient diffracting volumes. Initial macromolecule crystallization screening however often results in microcrystals, needle-like structures, needle clusters and/or inhomogeneous crystals with limited X-ray diffracting power. While extensive fine screening of buffer conditions may be used to obtain large single crystals, this is by no means a certain outcome. The development of synchrotron microfocus beamlines has allowed the collection of X-ray diffraction data suitable for structure determination from microcrystals and imperfect crystals (Sanishvili & Fischetti, 2017 ▸). Recent technological advances in X-ray crystallography [notably the development of serial femtosecond nanocrystallography (SFX) and the emergence of X-ray free-electron lasers (XFELs)] have opened new possibilities for collecting X-ray diffraction data from submicrometre protein crystals (Martin-Garcia *et al.*, 2016 ▸; Grünbein *et al.*, 2018 ▸; Owen *et al.*, 2017 ▸;Liu & Lee, 2019 ▸). For these techniques, a continuous flow of thousands of microcrystals is directed into the path of the X-ray beam and individual diffraction frames are collected from randomly oriented crystals (Martiel *et al.*, 2019 ▸; Chapman *et al.*, 2011 ▸). Nonetheless, the production of a jet of microcrystals is technically challenging, frames from several thousand submicrometre crystals must be collected, scaled and merged together to generate a complete data set (Beale *et al.*, 2019 ▸; Darmanin *et al.*, 2016 ▸), and widespread adoption of these techniques is hindered by limited access to relevant facilities (Liu & Lee, 2019 ▸).

As electrons interact with matter far more strongly than X-rays, in-house electron diffraction (ED) represents a promising alternative. Indeed, ED of unstained submicrometre two-dimensional crystals was used to elucidate the first three-dimensional model of a membrane protein (bacterio­rhodopsin) half a century ago (Henderson & Unwin, 1975 ▸). Indexing of ED patterns from three-dimensional crystals is however complicated due to the short (picometres) electron wavelength, which results in a flat Ewald sphere (Fultz & Howe, 2002 ▸). This has been mitigated through the development of three-dimensional electron diffraction (3D-ED) techniques (Kolb *et al.*, 2007 ▸; Shi *et al.*, 2013 ▸; Gemmi *et al.*, 2019 ▸), particularly the implementation of continuous rotation methods, known as microcrystal electron diffraction (MicroED; Nannenga, Shi, Leslie *et al.*, 2014 ▸) or continuous rotation electron diffraction (cRED; Cichocka *et al.*, 2018 ▸). The high electron-scattering cross section can however lead to (i) substantial inelastic scattering, which lowers the signal-to-noise ratio of the diffracted data, and (ii) multiple scattering events (‘dynamical diffraction’) from successive layers of the crystal, which complicates the relationship between the Coulomb potential density and the acquired intensities. To reduce both effects, thin (≲400 nm) crystals are preferred for ED analyses (Hattne *et al.*, 2015 ▸).

Substantial progress in 3D-ED/MicroED over the past decade (Clabbers & Abrahams, 2018 ▸) has allowed the determination of protein structures from crystals of micrometre and submicrometre dimensions (Zhang *et al.*, 2010 ▸; Nannenga, Shi, Hattne *et al.*, 2014 ▸; Gemmi *et al.*, 2019 ▸; Shi *et al.*, 2013 ▸), with more than a hundred protein structures now deposited in the Protein Data Bank (PDB; https://www.rcsb.org/stats). Most of these depositions however correspond to model proteins that have been studied to explore the potential and limitations of 3D-ED/MicroED as well as for methodological developments (Hattne *et al.*, 2018 ▸; Shi & Huang, 2022 ▸; de la Cruz *et al.*, 2017 ▸; Zhao *et al.*, 2021 ▸; Xu *et al.*, 2018 ▸; Lanza *et al.*, 2019 ▸; Yonekura *et al.*, 2019 ▸; Zhou, Luo, Luo *et al.*, 2019 ▸; Martynowycz *et al.*, 2019 ▸, 2020 ▸; Bücker *et al.*, 2020 ▸; Richards *et al.*, 2020 ▸; Blum *et al.*, 2021 ▸; Clabbers *et al.*, 2017 ▸). Only in 2019 was the first novel protein structure, that of the metalloenzyme R2lox, determined by 3D-ED/MicroED (Xu *et al.*, 2019 ▸), which was achieved by merging data collected from 21 crystals to 3 Å resolution with a completeness (the ratio between the number of measured and theoretically observable reflections) of 62.8%.

In a 3D-ED/MicroED analysis of biological macromolecules, small micrometre- or nanometre-sized crystals (microcrystals) are prepared by applying and vitrifying the sample on an EM grid, from which diffraction data are collected while rotating the sample stage continuously under a low-dose-rate electron beam (Nannenga, Shi, Hattne *et al.*, 2014 ▸). Recorded diffraction images are processed using conventional X-ray crystallography data-processing software, and Fourier transformation of the phased data set yields a Coulomb potential map that can be used for model building (Nannenga, 2020 ▸; Clabbers *et al.*, 2022 ▸). A major limitation of the method is the accumulation of radiation damage over the course of data acquisition (Xu *et al.*, 2018 ▸; Hattne *et al.*, 2018 ▸), which has an adverse effect on data quality, resolution and completeness. Reduced data completeness, which can greatly impact map quality and model accuracy (Nannenga & Gonen, 2014 ▸), also results from the limited rotation range of transmission electron microscope (TEM) stages, and can be further affected by crystal location on the grid, sample quality and crystal-specific characteristics such as shape and symmetry. While merging data from multiple randomly oriented crystals can be employed to enhance data completeness (Ge *et al.*, 2021 ▸; Wennmacher *et al.*, 2019 ▸; Bücker *et al.*, 2020 ▸; Xu *et al.*, 2018 ▸), this demands a high number of diffracting crystals. Serial electron crystallography (Serial-ED), whereby single ED patterns are collected from randomly oriented crystals on a sample grid at a fixed tilt angle, can address some of these issues (Smeets *et al.*, 2018 ▸; Bücker *et al.*, 2020 ▸), although (i) it is in general not possible to index protein diffraction data from a single frame due to the flatness of the Ewald sphere, necessitating prior knowledge of the lattice parameters for data processing, and (ii) processing a complete 3D-ED/MicroED data set is dependent on merging data from thousands of crystals. The latter is particularly problematic in cases where the crystals are heterogeneous in their packing (‘non-isomorphism’).

Thus, there is a need for data-acquisition strategies that can be applied to samples with a small number of diffracting crystals. This paper presents such an optimized data-acquisition and processing strategy for the collection of high-quality electron diffraction data from extended crystals using conventional cryo-electron microscopy (cryo-EM) instrumentation. Our approach, inspired by helical data-acquisition schemes employed in X-ray crystallography (Flot *et al.*, 2010 ▸; Polsinelli *et al.*, 2017 ▸), yields both high data completeness and redundancy. This is achieved by collecting and merging angular segments of ED data from noncontiguous regions of a single crystal, which are then merged with corresponding data from other differently oriented crystals. This multi-position acquisition strategy provides an effective means of reducing the effects of non-isomorphism while minimizing radiation damage, allowing the acquisition of complete electron diffraction data from beam-sensitive crystals. We have applied this method to solve the structure of the C-terminal peptide (amino acids 617–684) of human Grb2-associated binding protein 1 (Gab1^617–684^) in complex with the N-terminal region (amino acids 1–222) of the tyrosine-protein phosphatase non-receptor type 11 (SHP2^1–222^) (Machner *et al.*, 2026 ▸). We also discuss here the challenges posed by sample preparation and data processing for structure determination as well as our solutions, which can be further fine-tuned for other samples on a case-by-case basis.

## 2. Materials and methods

### 2.1. Sample preparation

**Figure 1:**
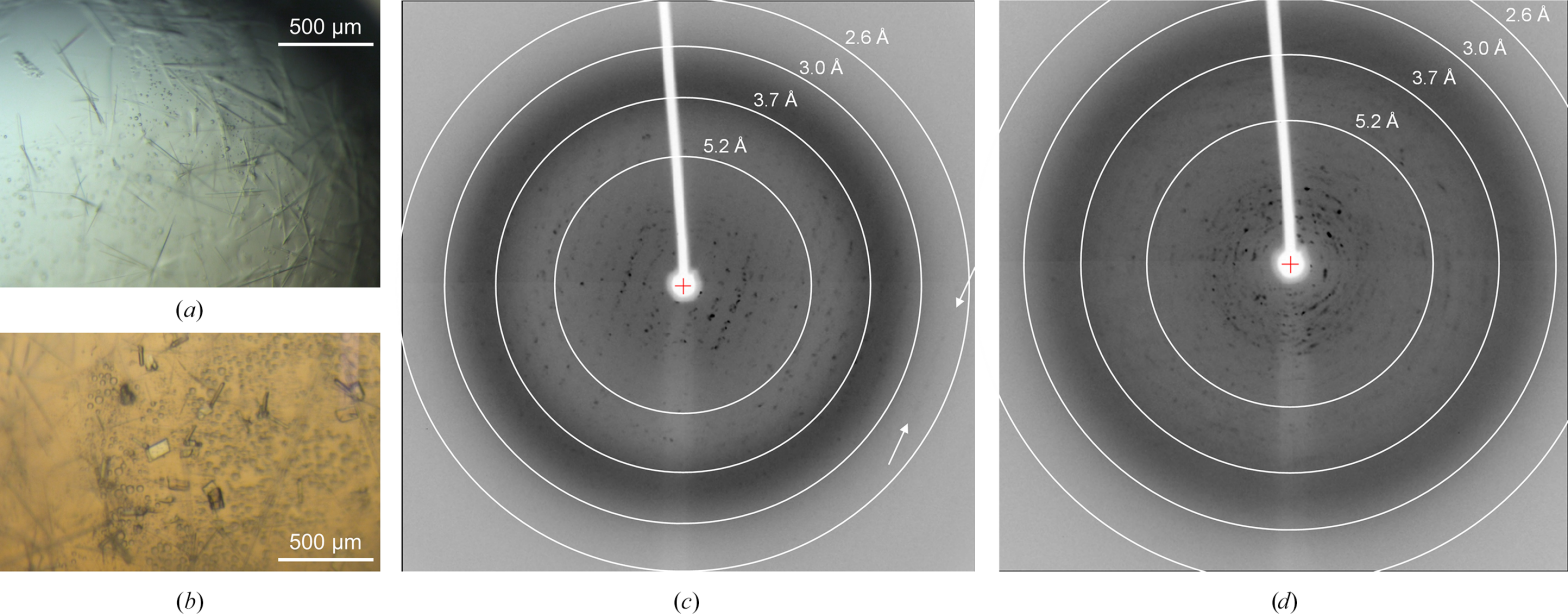
Macroscopic crystals of the Gab1–SHP2 complex demonstrate disorder. (*a*) Light microscope view of needle-like Gab1–SHP2 crystals grown in a hanging drop using the vapor-diffusion method. (*b*) After 18 days, small plate-like crystals grew in the same drop. (*c*, *d*) X-ray diffraction patterns of two independent plate-like crystals (*b*) reveal Bragg reflections up to around 2.7 Å resolution (white arrows), but exhibit multiple lattices and high mosaicity.

Protein expression and purification of SHP2^1–222^ and the intrinsically disordered C-terminal part of Gab1^617–684^ phosphorylated at Tyr627 and Tyr659 are described elsewhere (Machner *et al.*, 2026 ▸). Prior to crystallization, SHP2^1–222^ (25 mg ml^−1^) was mixed with Gab1^617–684^ (7.7 mg ml^−1^) in 20 m*M* bis-Tris pH 6.5, 50 m*M* NaCl, 1 m*M* DTT. The complex was crystallized by hanging-drop vapor diffusion in 15-well EasyXtal crystallization plates (Qiagen). Thin needle-shaped crystals appeared in 0.1 *M* Tris pH 8.8, 29% PEG 3350 within seven days at 12°C (Fig. 1 ▸*a*), which were followed after another ten days by more compact plate-like crystals (Fig. 1 ▸*b*).

**Figure 2:**
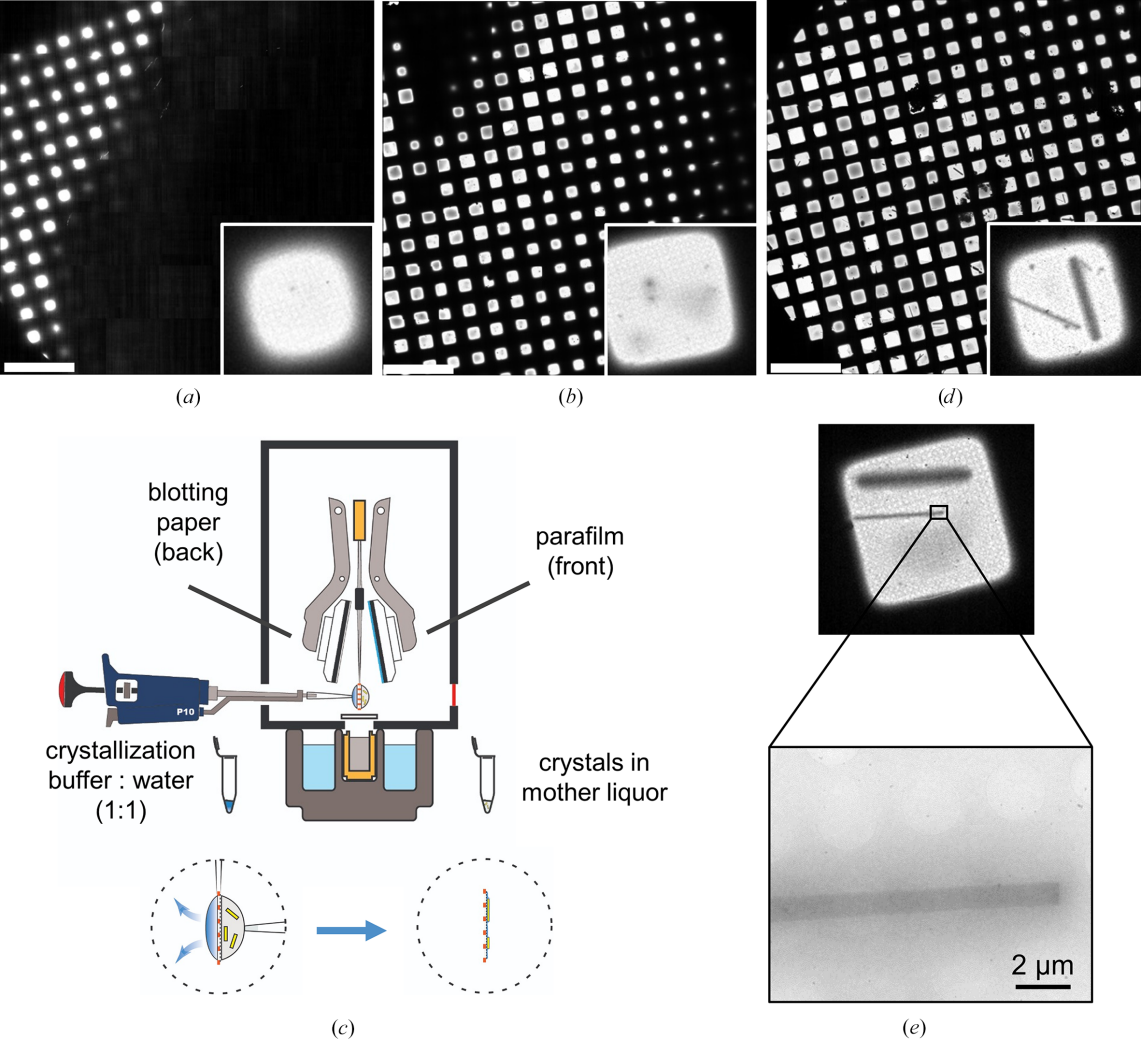
Procedure for vitrification of Gab1–SHP2 complex crystals using the ThermoFisher Vitrobot Mark IV. (*a*, *b*, *d*) All-grid atlas views of crystal specimens (scale bar 450 µm) with enlarged views of corresponding single grid squares (insets, box edges 150 µm). (*a*) Gab1–SHP2 crystal sample (viscous slurry containing 29% PEG 3350) by double-sided blotting for 12 s at 4°C and 95% humidity. (*b*) Gab1–SHP2 sample upon increasing the double-sided blotting time to 24 s. (*c*) Schematic drawing demonstrating the optimized vitrification setup for preparing protein crystals for electron diffraction experiments. Complex crystals were harvested from the crystallization drops and applied directly to the front face of the EM grid. Crystallization buffer, diluted 1:1 with water, was applied to the back face of the grid. The plunger was mounted with a Parafilm disk on the side facing the crystals and with blotting paper on the side facing the buffer solution. (*d*) Gab1–SHP2 specimen prepared using the optimized setup, revealing microcrystals on the grid. (*e*) Low-magnification cryo-EM image of Gab1–SHP2 complex crystals observed after sample vitrification using the optimized setup. Two long rod-shaped crystals are visible on the grid.

For 3D-ED/MicroED, a skin that formed at the surface of the drop was removed and the entire remaining drop including the mother liquor and crystals was transferred to a 1.5 ml tube. Crystals were pooled from several crystallization drops and used directly to make vitrified samples. Quantifoil R2/1 grids, plasma-treated in a PELCO easiGLOW glow-discharging machine at 15 mA for 25 s under 0.4 mbar residual air pressure, were mounted in a ThermoFisher Scientific Vitrobot Mark IV (which is designed to blot both sides simultaneously; front and back) with the chamber temperature and humidity adjusted to 4°C and >95%, respectively. Initial attempts to vitrify the Gab1–SHP2 crystals following protocols developed for lysozyme (Bücker *et al.*, 2020 ▸) resulted in grids with thick ice (see Fig. 2 ▸*a*), presumably due to the viscous 29% PEG 3350 in the drops. Sample vitrification was therefore modified as outlined below in Section 3 (see Figs. 2 ▸*b*–2 ▸*d*). The filter paper facing the sample (front face) was replaced by a similarly cut disk of clean and unstretched Parafilm and a 3.5 µl aliquot of the crystal-containing suspension was applied to the carbon film side of the grid (front face). 3.5 µl reservoir solution mixed with water (1:1 ratio) was applied to the other (back) face of the grid facing a 595-grade ashless filter-paper pad, and the grid was blotted for 25 s, so that the excess liquid was absorbed from the back face and caused the crystals to settle on the carbon face. All prepared grids were plunged into liquid ethane, clipped in an autogrid assembly and loaded in the electron microscope under cryogenic conditions (liquid-nitrogen temperature).

### 2.2. X-ray diffraction

Crystals were flash-cooled in liquid nitrogen with 10% ethylene glycol added as a cryoprotectant. Diffraction patterns were collected in-house at 100 K with Cu *K*α radiation (λ = 1.5418 Å) using a CCD detector (Saturn 944++, Rigaku/MSC, Tokyo, Japan) mounted on a rotating-anode generator (MicroMax-007, Rigaku/MSC, Tokyo, Japan).

### 2.3. Electron diffraction data acquisition

#### 2.3.1. Electron microscope configuration and adjustments

Electron diffraction experiments were conducted using a 200 keV Thermo Scientific Glacios cryogenic transmission electron microscope equipped with a Ceta-D camera and a Falcon 4i direct electron camera. Throughout our experiments, both the microscope stage and the autoloader were maintained at cryogenic temperatures (<100 K). Microscope alignments and calibration are described in detail in Supplementary Section S1.

#### 2.3.2. Electron diffraction data collection

Continuous rotation ED data were collected using the *EPU-D* software (version 1.11), with a 50 µm C2 aperture to limit the beam size to 1.7 µm in the parallel beam nanoprobe. Prior to acquiring diffraction patterns from a crystal, the eucentric height (EH) was readjusted. Since the optimal fluence limit of the electron diffraction data is very small (Martynowycz *et al.*, 2022 ▸), we decided to adjust the EH with a minimal possible incident beam fluence by adjusting the height at a nearby location rather than on the region of interest (ROI) of the crystal. One of the following methods was used for such low-fluence EH adjustments.

(i) If crystals were evenly distributed and well separated on the grid and the grid square appeared flat and undamaged, a suitable empty spot for adjusting the EH was easily located using standard procedures. In such instances, the stage was moved to navigate to a vacant area nearby (∼15 µm) and the EH was fine-tuned in the Search/Auto-Eucentric preset of the *EPU-D* software using the Auto-Eucentric Height function.
(ii) In cases where approach (i) was not feasible, for example due to numerous crystals, contaminations near the ROI or difficulties arising from thick ice, the EH was adapted at a higher magnification and a smaller beam diameter, essentially the imaging preset. To achieve this, the objective lens was set to the calibrated Eucentric-Focus value as described in Supplementary Section S1, and the *Z*-stage movement was used to focus the region >2 µm away from the edge of the crystal.

In both scenarios, the EH adjustment was checked by capturing very low-fluence and low-magnification images of the targeted crystal region with a ±30° tilt (or at the maximum tilt range if this was larger) to ensure that the crystal remained in the field of view and nothing obstructed the beam during the tilt.

Following these adjustments, the stage was navigated again to the grid position used to make the EH adjustment (empty carbon film). The microscope was set to diffraction mode, and the direct [000] beam position was determined on the fluorescent screen. This allowed precise placement of the central beam under the beam-stopper to conceal it, preventing camera saturation and pixel blooming, while preserving the clarity of fine diffracting spots. The beam was blanked and the stage was adjusted back mechanically to position the ROI of the crystal under the beam, with no changes to the optical adjustments. After unblanking the beam, data recording was started immediately to minimize any potential beam drifts.

For the elongated Gab1–SHP2 complex whiskers, multiple acquisitions were recorded along different sections of a single crystal (resulting in multiple diffraction data sets for each crystal), thereby minimizing the cumulative radiation damage to maintain a high signal-to-noise ratio. When possible, parts of the crystal located in the grid holes were preferred to avoid the background contribution of the grid film material. After collecting one segment of data, the stage was moved along the crystal, leaving at least 1 µm between the edges of the recording areas to avoid overlapping the exposure areas. Depending on the length, diffraction quality and grid position of the crystal, up to 15 data sets with 20–30° tilt segments (0.5° per frame) were acquired to cover the maximum possible tilt range of the sample stage (−70° to 70°) in overlapping tilt intervals (Table 1 ▸, Supplementary Table S2). A single acquisition with a higher tilt range of 60° (−30° to 30°) was recorded around the center of the tilt axis to facilitate determination of the space group and unit-cell parameters. High-tilt-angle segments (−70° to −50° and 50° to 70°, respectively) were collected multiple times, as several such segments could not be processed due to high background noise (resulting from the increased sample/ice thickness at high angles and/or beam-path obstruction) and had to be excluded from the final data set. The incident beam fluence during data collection was fixed and the data were collected with a total cumulative fluence of 2–4 e^−^ Å^−2^, except for the acquisition with the higher tilt range, which was collected with a total cumulative fluence of around 5 e^−^ Å^−2^. Electron diffraction images were recorded as single frames in SMV format.

### 2.4. Data processing and model building

Data processing was carried out using the *XDS* software package (Kabsch, 2010 ▸). The orthorhombic space group *P*2_1_2_1_2_1_ was identified from the central tilt range (−30° to 30°) with unit-cell parameters *a* = 30.51, *b* = 82.15, *c* = 118.16 Å, α = β = γ = 90°. These values were subsequently used to process data sets acquired at other tilt ranges. Data sets collected at multiple tilt ranges along the same crystal were merged in *XSCALE* (Kabsch, 2010 ▸), and were merged with the corresponding data from isomorphous crystals (unit-cell parameter differences of less than 1%). Only individual tilt segments that contributed to improved quality (CC_1/2_), redundancy and/or completeness of the merged and scaled data were included in the final data set (Karplus & Diederichs, 2012 ▸). Diffraction data to a resolution of 3.2 Å with a completeness of 89% could be obtained from just two crystals (Table 1 ▸), with averaged unit-cell parameters *a* = 30.59, *b* = 82.25, *c* = 117.76 Å, α = β = γ = 90° calculated in *CELLPARM* implemented in *XSCALE*. Merged data were exported in MTZ format using *XDSCONV* (Kabsch, 2010 ▸) and initial phases were determined by molecular replacement with *Phaser* (McCoy *et al.*, 2007 ▸) using the crystal structure of SHP2^1–222^ (PDB entry 5df6, chain *A*; Liu *et al.*, 2016 ▸) as the search model. The search model was split into two domains, with domain 1 consisting of residues 4–104 (TFZ score 23.1) and domain 2 consisting of residues 108–220 (TFZ score 10.4). The molecular-replacement solution was initially refined in *REFMAC* (Murshudov *et al.*, 2011 ▸) from the *CCP*4 software package (Agirre *et al.*, 2023 ▸) using scattering factors for electrons, derived from X-ray form factors using the Mott–Bethe approximation. The Coulomb potential map revealed density for the SHP2 linker between the two domains as well as residues of the bisphosphorylated Gab1 peptide, which were fitted to the density using *Coot* (Emsley & Cowtan, 2004 ▸) and refined with *Phenix* (Liebschner *et al.*, 2019 ▸) to 3.2 Å resolution. Refinement statistics for the final model, comprising SHP2 residues 6–221 and two Gab1 fragments (624–634 and 653–672), are summarized in Supplementary Table S3. Coordinates and structure factors have been deposited in the Protein Data Bank (PDB entry 9qcd). Figures were prepared using *PyMOL* (version 2.0; Schrödinger).

## 3. Results

The Gab1–SHP2 complex crystallized as very thin whisker-like crystals (Fig. 1 ▸*a*) that did not diffract X-rays in-house, probably due to their small volume. Plate-like crystals that grew in the same drops (Fig. 1 ▸*b*) did diffract X-rays to up to 2.7 Å resolution, but exhibited considerable disorder, with multiple lattices (Figs. 1 ▸*c* and 1 ▸*d*). 3D-ED/MicroED was therefore explored as a promising alternative for structure determination, given its potential to collect data from very small crystal volumes. Initial electron diffraction experiments utilizing our in-house 200 kV instrument were performed following published protocols for lysozyme (Bücker *et al.*, 2020 ▸; de la Cruz *et al.*, 2017 ▸; Xu *et al.*, 2018 ▸; Nannenga, Shi, Leslie *et al.*, 2014 ▸; Shi *et al.*, 2013 ▸). Vitrification according to procedures for lysozyme crystals (Bücker *et al.*, 2020 ▸), *i.e.* fragmentation of the Gab1–SHP2 crystals by sonication followed by double-sided blotting for 12 s, resulted in grids with thick ice that obscured the majority of the grid squares (Fig. 2 ▸*a*). The few grid squares visible within the blotting area exhibited rounded edges, indicating relatively thick ice even in the visible area. Notably, the Gab1–SHP2 complex crystallized in 29% PEG 3350, resulting in a highly viscous sample solution. Increasing the blotting time to 24 s resulted in a non-uniform gradient of vitrified ice thickness throughout the sample, with more visible grid squares (Fig. 2 ▸*b*). Although grid holes could be seen, indicating areas with a thinner ice layer, no protein crystals could be identified in the blotted sample. To test whether microcrystals were damaged during sonication, crystal fragmentation was tried using a glass rod and vortexing with seed beads. No crystals were identified in any of the prepared samples. To avoid the possibility that crystals were lost during the fragmentation process, crystal suspensions were pooled from the hanging drops and applied directly to the grid without any further treatment. Once again, no crystals were observed in any of the grid squares.

**Figure 3:**
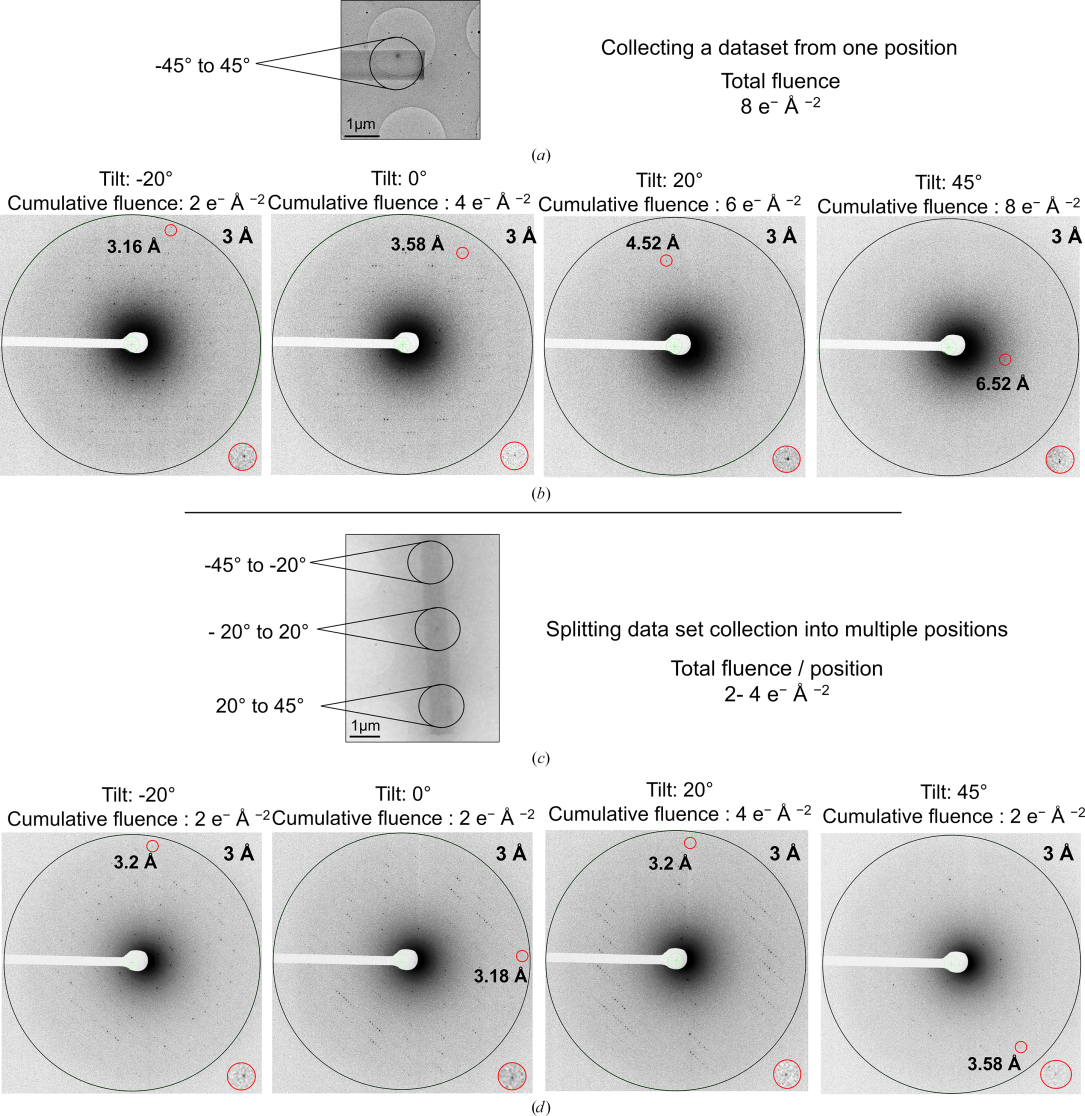
A multi-position data-acquisition strategy reduces the cumulative incident beam fluence during 3D-ED/MicroED data collection. (*a*) A crystal used for 3D-ED/MicroED data collection at a single position while tilting the sample stage through a total range of 90°. (*b*) The resulting diffraction patterns at the indicated tilt angles with the corresponding cumulative incident beam fluence. (*c*) Multi-position 3D-ED/MicroED data collection involves the acquisition of diffraction data from multiple positions along a single crystal within an accumulated total tilt range of 90°. (*d*) The resulting diffraction patterns taken at the indicated tilt angles using a cumulative fluence of ≤4 e^−^ Å^−2^ throughout the acquisition range. For each diffraction image, the highest resolution Bragg reflection is highlighted with a red circle (magnified view on the lower right). Data recorded using the multi-position strategy show significantly improved diffraction, with distinct reflections up to around 3.2 Å resolution.

To investigate whether microcrystals may have been lost due to adhesion to the filter paper, back-face blotting was explored (Clabbers *et al.*, 2022 ▸; Tan & Rubinstein, 2020 ▸). As the Vitrobot does not provide this possibility directly, the front-face filter paper was replaced with an unstretched piece of Parafilm that was cut to match the size and shape of the filter paper (Fig. 2 ▸*c*). Initial attempts at back-face blotting resulted in an opaque sample, indicating thick ice. A 3.5 µl drop of crystallization buffer diluted 1:1 with water was applied to the back face of the grid, with the reasoning that this could establish a connection between the viscous mother liquor of the crystals and the filter paper to allow efficient withdrawal of excess liquid through the sample grid holes. Blotting for 25 s resulted in a gradient of thin ice across the sample grid (Fig. 2 ▸*d*) in which most grid squares were clearly visible with sharp and faceted edges, especially at the center of the grid and in the direction of thinner ice. In particular, thin crystals ranging in width from 0.5 µm to a few micrometres and of diverse lengths were observed across the sample grid at various magnifications, with some extending along several grid holes and even across several grid squares (Figs. 2 ▸*d* and 2 ▸*e*). The grid was screened to select crystals with a thickness suitable for electron diffraction data collection, several of which proved promising in diffraction mode (Fig. 3 ▸*b*). Suitable ice thickness with visible crystals was achieved only when both back-face blotting and addition of diluted buffer were performed during sample preparation.

Following optimization of sample preparation, crystals were screened for diffraction potential. The cryo-EM system used here operates at 200 kV, which results in a higher stopping power and inelastic scattering cross sections compared with 300 kV and therefore necessitates thinner specimens (Berger & Seltzer, 1983 ▸). The crystals were screened for diffraction behavior by recording single diffraction images without tilting the sample stage (tilt angle 0°) using a fluence of ∼0.1 e^−^ Å^−2^. For crystals diffracting to a resolution of 3.5 Å or higher, data sets were collected within a total tilt range of 90° (−45° to 45°) using a total fluence of 8 e^−^ Å^−2^ (Fig. 3 ▸*a*). Rapid crystal deterioration was observed as the acquisition progressed, indicated by a rapid decrease in the resolution limit on consecutive frames (Fig. 3 ▸*b*). Frames collected to a cumulative fluence of ≲4 e^−^ Å^−2^ revealed Bragg reflections to resolutions higher than 3.6 Å. Thereafter, the number of observed diffraction spots per frame decreased rapidly, with the resolution limit falling to ∼4.5 Å at a cumulative fluence of 6 e^−^ Å^−2^ and to ∼6.5 Å at a cumulative fluence of 8 e^−^ Å^−2^ (Fig. 3 ▸*b*). Subsequent experiments showed that larger angular ranges than anticipated (>20°; Nannenga & Gonen, 2019 ▸) were required for indexing; at the initial fluences applied, we assume that the reduction in the number of reflections in the chosen angular range, as well as possible changes in the cell parameters, due to radiation damage accrued during exposure precluded indexing and thereby further processing.

To minimize the cumulative incident beam fluence applied to the crystal during acquisition, two modifications were implemented to the data-collection procedure. Firstly, eucentric height adjustment prior to diffraction data collection was performed near the region of interest with a minimal fluence, avoiding exposing the crystal itself. Secondly, we took advantage of the elongated rod-like shape of the crystals. Instead of recording data from a single position, frames from consecutive tilt ranges were split over several positions along an individual crystal (Fig. 3 ▸*c*), inspired by helical X-ray diffraction data-acquisition strategies for needle-like crystals at synchrotron facilities (Flot *et al.*, 2010 ▸; Polsinelli *et al.*, 2017 ▸). Specifically, the tilt range was divided into three intervals, −45° to −20, −20° to 20° and 20° to 45° (Fig. 3 ▸*c*), with a wider tilt window around the zero-tilt angle to aid in indexing. Collecting fewer frames over smaller tilt ranges resulted in a reduction in beam exposure of the illuminated section of the crystal (and thereby radiation damage). Combining tilt series from different parts of the same crystal maximizes reciprocal-space coverage while minimizing the effective total exposure over the entire acquired tilt range, resulting in data collected with a total cumulative fluence of 2–4 e^−^ Å^−2^. This is a reduction in the total applied incident beam fluence by at least one half (Fig. 3 ▸*d*), maintaining the diffraction quality of the crystal (∼3.2 Å) across the entire applied tilt range.

**Figure 4:**
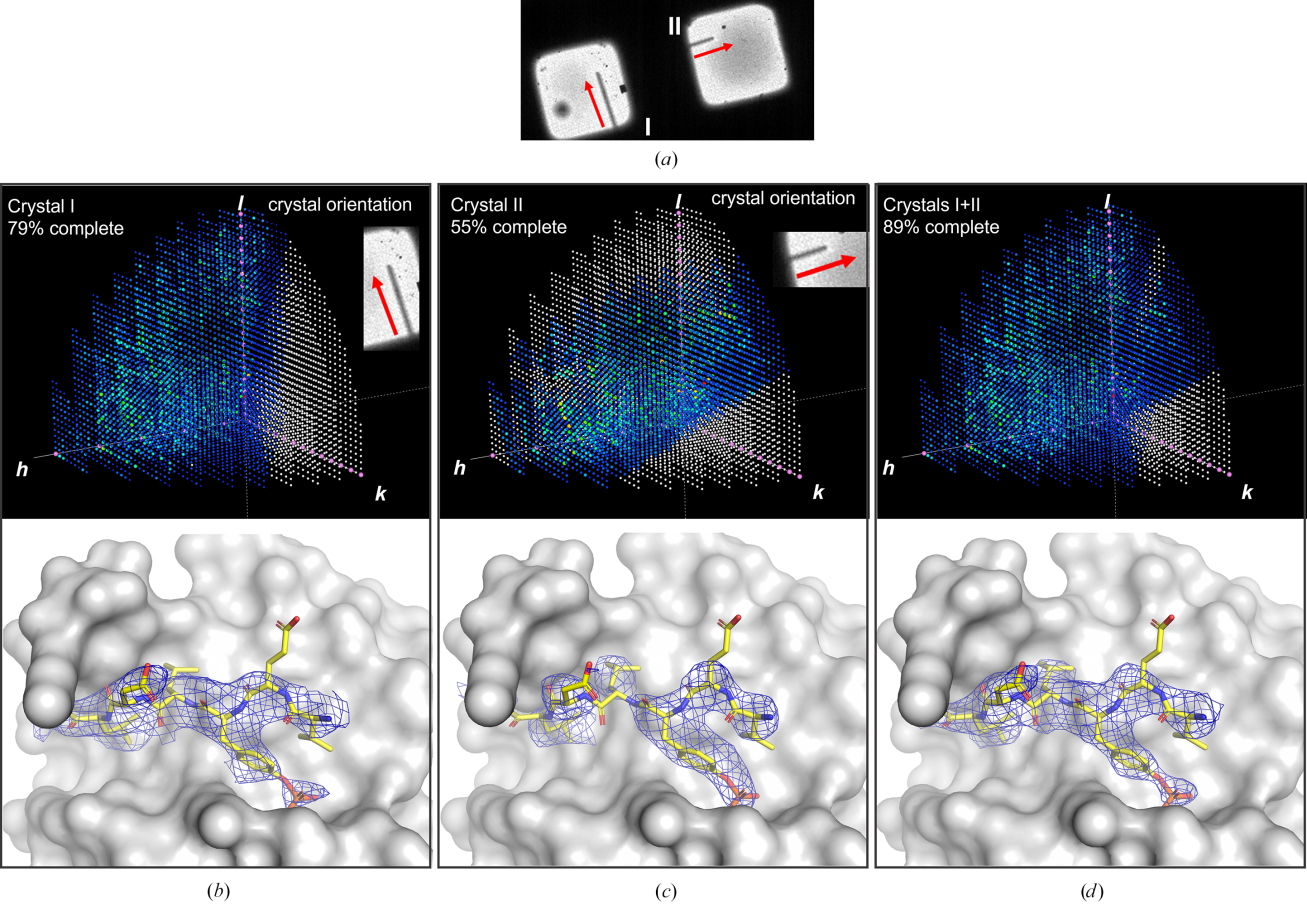
Improving data completeness by merging data collected from crystals in different orientations on the sample grid. (*a*) Enlarged atlas view showing crystals I and II positioned perpendicular to each other on adjacent grid squares of the same sample. The EM stage tilt axis is vertical, and red arrows show the direction of consecutive data-acquisition regions. (*b*, *c*, *d*) Effect of data completeness on the quality of calculated maps for the Gab1 fragment. Upper panels: reciprocal-space reflection distribution prepared with 3*D Data Viewer* in *Phenix* (Liebschner *et al.*, 2019 ▸). Missing reflections are shown in white and systematic absences in pink; it is evident from the former that the b* axes of both crystals are in the direction of the electron beam. Lower panels: 2*F*_o_ − *F*_c_ maps (contoured at 1.5σ, blue mesh) for Gab1 residues 624–631 (yellow sticks) in complex with the N-terminal domain of SHP2^1–222^ (gray surface) using phases from the final structure refined against the corresponding data set. (*b*) Data collected from crystal I (oriented perpendicular to the rotation axis) with a total completeness of 79% results in density for the peptide but misses a significant wedge of data. (*c*) Crystal II (oriented parallel to the rotation axis) yielded data with a completeness of 55%, resulting in recognisable yet broken density for the peptide. (*d*) Merging the data from both crystals I and II significantly reduces the missing wedge, resulting in a better-defined density with improved connectivity.

The electron diffraction data could be indexed in the orthorhombic space group *P*2_1_2_1_2_1_. Combining three segments collected from a single crystal over a total tilt range of 90° yielded data to a resolution of 3.2 Å with a completeness of 69% (Supplementary Table S2). The corresponding data from a second crystal were 61% complete, but turned out to represent the same region of reciprocal space (Supplementary Fig. S2), indicating a similar orientation on the grid. In order to increase reciprocal-space completeness systematically, data were collected from crystals positioned in orthogonal orientations on the grid, allowing the individual crystals to be tilted around different crystallographic axes. In addition, we utilized the maximum allowed sample-stage tilt range (−70° to 70°). The latter can be performed only when the crystal has a clear path to the beam at high tilt angles that is not obstructed by grid bars, other crystals or contamination. Further screening of the grids identified two adjacent crystals, I and II, that were positioned perpendicular to one other (Fig. 4 ▸*a*). Due to the length of crystal I (oriented nearly perpendicular to the tilt axis), data collection could be performed several times in overlapping tilt intervals, increasing the quality and redundancy of the data. Only segments exhibiting isomorphous characteristics that could be processed to reasonable statistics were merged (as explained in Section 2), resulting in a combined angular range of −30° to 70° for crystal I (Table 1 ▸). Merged segments from this crystal achieved a completeness of 79% (predicted to be 82.6% for the applied tilt ranges using *iMOSFLM*; Battye *et al.*, 2011 ▸) at 3.2 Å resolution (Fig. 4 ▸*b*, Table 1 ▸). For crystal II, which was oriented almost parallel to the tilt axis, high-tilt data were collected from three separate positions. These covered angular ranges from −68° to −50° and 30° to 64°, yielding a data set with 55% completeness (predicted 65%) at 3.2 Å resolution (Fig. 4 ▸*c*). These lower than predicted values of completeness demonstrate the effects of experimental factors such as the low signal-to-noise ratio of the detector, the accumulation of radiation damage, crystal imperfections, the position of the crystal relative to the tilt axis and the decrease in peak intensity at high tilt angles. Merging the data from both crystals resulted in an overall completeness of 89% at 3.2 Å resolution, with 〈*I*/σ(*I*)〉 and CC_1/2_ values of 3.29 and 94%, respectively (1.4 and 53%, respectively, in the highest resolution shell; Table 1 ▸, Fig. 4 ▸*d*). Coulomb potential maps calculated from the merged data set are of superior quality, with better connectivity compared with the maps calculated from each crystal separately (Fig. 4 ▸), displaying clear density for the Gab1 fragment bound to SHP2^1–222^ (Fig. 4 ▸*d*, Supplementary Fig. S3). The biological function and significance of the structure are presented elsewhere (Machner *et al.*, 2026 ▸).

## 4. Discussion

The procedures outlined in this paper were essential to determine the structure of the Gab1–SHP2 complex (Machner *et al.*, 2026 ▸). Although the crystals obtained were unsuitable for X-ray diffraction studies, either due to their thin needle-like form (yielding insufficient diffraction volume) or through crystal defects (splitting, twinning and/or high mosaicity) (Fig. 1 ▸), they proved to be amenable to electron diffraction. Obtaining submicrometre-thick crystals embedded in a thin layer of vitrified ice (Mu *et al.*, 2021 ▸) is essential to 3D-ED/MicroED as this allows electrons to penetrate the sample with minimal inelastic scattering (Martynowycz *et al.*, 2019 ▸). Microcrystals can be grown by adjusting the crystallization conditions (Russo Krauss *et al.*, 2013 ▸; McPherson & Gavira, 2014 ▸) or obtained through the fragmentation of larger crystals by, for example, crushing with a glass rod, vortexing with beads or treatment in a sonication bath (de la Cruz *et al.*, 2017 ▸; Bücker *et al.*, 2020 ▸). In the present case, microcrystals could be harvested directly from drops that contained macroscopic crystals without further preparation steps. Cryogenic focused ion beam (cryo-FIB) milling (Nicolas, Gillman *et al.*, 2025 ▸; Shaikhqasem & Stubbs, 2024 ▸) provides an alternative route to preparing crystals of ideal thickness, even for such challenging crystals as those obtained using lipidic cubic phases (LCP; Duyvesteyn *et al.*, 2018 ▸; Zhou, Luo & Li, 2019 ▸; Martynowycz & Gonen, 2021 ▸; Beale *et al.*, 2020 ▸; Martynowycz *et al.*, 2019 ▸); on the other hand, cryo-FIB milling is not readily available in cryo-EM laboratories due to costly specialized instrumentation.

In the present study, it was necessary to adopt a single-sided back-blotting procedure prior to vitrification in order to obtain crystals on the EM grid, a method that also proved to be crucial for the vitrification of R2lox crystals (Xu *et al.*, 2019 ▸), which similarly grew in a viscous (44% PEG 400) mother liquor. The simple modifications to the blotting setup introduced here (Fig. 2 ▸*d*) allow the vitrification of crystals grown in high-viscosity buffers under controlled conditions of temperature and humidity. Another promising approach is the recently introduced pressure-assisted method, Preassis (Zhao *et al.*, 2021 ▸), which uses suction pressure to draw excess liquid through a sample grid resting on filter paper.

The crystals showed rapid deterioration even at relatively low beam fluence (radiation per unit area in e^−^ Å^−2^; Fig. 3 ▸). It should be noted that radiation damage is dependent upon the irradiated volume and not the area (Holton, 2009 ▸), but as we were unable to measure crystal thicknesses, fluence is used as a measurable proxy. It has been shown that electron-induced site-specific damage can occur at incident beam fluences as low as ∼0.9 e^−^ Å^−2^ (disulfide-bond cleavage) or ∼2.5 e^−^ Å^−2^ (decarboxylation of acidic side chains) (Hattne *et al.*, 2018 ▸). Application of the multi-position acquisition strategy reduced the cumulative incident beam fluence by half, enabling data collection as multiple tilt segments with continuous rotation. This provides more efficient data collection than, for example, *SerialED* (Smeets *et al.*, 2018 ▸; Bücker *et al.*, 2020 ▸), yielding a higher coverage of reciprocal space per position and crystal, as well as providing a sufficient wedge of data for indexing uncharacterized crystals. A similar segment-wise approach has been used to collect ED data from thin small-molecule crystal needles (Gruene *et al.*, 2018 ▸), although small-molecule crystals tend to be less susceptible to radiation damage due to tighter packing and an absence of bulk solvent. The data-collection strategy implemented here is not limited to needle-like crystals, but can also be applied to other morphologies such as thin plates. It might also be possible to restrict the electron beam to smaller diameters, which would allow an increase in the number of exposure sites per crystal and could further improve data coverage, although this must be weighed up against a reduction of the exposed sample volume that would result in decreased reflection intensities. An alternative strategy has recently been proposed to address the missing-cone data-completeness issue (Gillman *et al.*, 2024 ▸): preferred crystal orientation is avoided by growing crystals directly on a grid without a support film (suspended drop crystallization), allowing them to adopt random orientations with respect to the beam. To the best of our knowledge, however, equipment for support-free crystallization is not generally available at the present time; moreover, such samples require additional preparation by cryo-FIB milling, which involves significant cost and specialized instrumentation that is not readily accessible to many laboratories.

Through a systematic choice of crystals with recognizably different orientations, a data set with 89% completeness could be obtained from analyzing just two Gab1–SHP2 crystals, compared with the thousands needed to obtain comparable completeness using *SerialED*. Nevertheless, some reflections remained inaccessible due to a preferred orientation of the crystals (each with the crystallographic b* axis perpendicular to the grid; Fig. 4 ▸, Supplementary Fig. S2). This could be alleviated through the use of a high-tilt-angle sample holder capable of 360° rotation, as developed recently for electron tomography (Kawase *et al.*, 2007 ▸; Kato *et al.*, 2008 ▸; Thermo Fisher Scientific, 2024 ▸).

As we were unable to index any of the X-ray diffraction patterns, it is not possible to determine whether the microcrystals used for ED data collection are the same form as the macroscopic needles or plates, only that the microcrystals diffract electrons. Hence, we are unable to say definitively whether the 3.2 Å resolution ED data set obtained here represents the ultimate resolution limit of these crystals. Nevertheless, the application of an energy filter (which would decrease background noise due to removal of inelastically scattered electrons; Yonekura *et al.*, 2002 ▸), together with replacement of the fiber optic coupled imaging sensor used here with either a direct electron detector (Hattne *et al.*, 2019 ▸) or a hybrid pixel detector (McMullan *et al.*, 2007 ▸; Tinti *et al.*, 2018 ▸), could increase the signal-to-noise ratio of the diffraction data and thereby the resolution.

## 5. Conclusions

Although the first protein structure from three-dimensional crystals was solved by ED over a decade ago (Shi *et al.*, 2013 ▸) and the first novel structure in 2019 (Xu *et al.*, 2019 ▸), electron protein crystallography has to date been practiced in only a handful of specialized laboratories. This is in stark contrast to macromolecular X-ray crystallography and to the mushrooming of laboratories equipped with cryo-electron microscopes suited to high-resolution structure determination. Transitioning from X-ray to electron crystallography is not straightforward for a number of reasons. The much stronger interaction of electrons with matter can result in multiple scattering events within the crystal (dynamical diffraction) which complicate the relationship between the diffraction intensities and the unit-cell contents (Klar *et al.*, 2023 ▸); this is alleviated through the use of thin specimens, which can still be problematic at high tilt angles. The more intense interaction also results in higher sensitivity to radiation damage, requiring low electron doses and cryogenic temperatures, with the latter also being necessary to preserve the sample in the EM column vacuum. Finally, the short (picometre) electron beam wavelength results in a flat and crowded Ewald sphere surface, posing potential problems for indexing.

Many of these aspects have been approached with instrument-based optimizations such as cryo-FIB milling (Duyvesteyn *et al.*, 2018 ▸) and the use of direct or hybrid pixel detectors (Hattne *et al.*, 2019 ▸; Tinti *et al.*, 2018 ▸), aided by the recently introduced method of suspended-drop crystallization (Gillman *et al.*, 2024 ▸), but these are often costly and not widely available. Even using such high-end facilities, the acquisition of a data set with sufficient completeness remains challenging (Nicolas, Shiriaeva *et al.*, 2025 ▸), with most protein 3D-ED/MicroED data collections relying on merging data from a large number of randomly oriented crystals. The strategies described here, inspired by classical X-ray crystallography approaches such as helical data collection and data acquisition about multiple rotation axes, are simple, applicable to a wide range of instruments and compatible with more advanced infrastructure. It should be noted that ED is not simply an extension of X-ray crystallography for determining structures, but is complementary to microcrystal X-ray difffraction (Tremlett *et al.*, 2025 ▸); ED yields an electrostatic potential distribution (in contrast to the electron-density distribution obtained from X-ray diffraction), raising the possibility of determining charge states at atomic resolution (Takaba *et al.*, 2023 ▸). With the ever-increasing availability of cryo-EM infrastructure, it is possible to envisage the application of 3D-ED/MicroED to the screening of crystallization setups containing microcrystal showers to identify conditions suitable for further optimization and, in favorable cases, collect ED data for structure determination. Based on the experience gained in these studies, it is to be hoped that the practice of 3D-ED/MicroED becomes more widespread in the macromolecular crystallo­graphy community to solve structures from crystals that have eluded analysis using classical methodologies.

## 6. Related literature

The following reference is cited in the supporting information for this article: Williams & Carter (2009 ▸).

## Supplementary Material

Supplementary Methods, Supplementary Figures and Supplementary Tables. DOI: 10.1107/S2059798325011258/he5693sup1.pdf

## Figures and Tables

**Table 1:** Data-collection statistics for crystals used to determine the structure of the Gab1–SHP2 complex (space group *P*2_1_2_1_2_1_; all data were collected at an accelerating voltage of 200 kV, corresponding to an electron wavelength of 0.025 Å) Data were collected on a Ceta-D detector at a nominal optical length (*i.e.* crystal-to-detector distance) of 1704 mm. Data were recorded with an oscillation range of 0.5° per frame. Values in parentheses are for the highest resolution shell.

Acquisition	Angular range collected (°)	Resolution (Å)	Total reflections	Total unique reflections	Completeness (%)	〈*I*/σ(*I*)〉	CC_1/2_ (%)	*R*_meas_ (%)	Mosaicity (°)
Data reduction for crystal I, positioned perpendicular to the rotation axis (*a* = 30.55, *b* = 81.85, *c* = 117.94 Å)									
1	30 to 70	28.0–3.5 (3.71–3.50)	5040 (779)	1884 (292)	45.6 (46.3)	1.73 (0.6)	78 (22)	118 (305)	0.75
2	10 to 30	28.7–3.2 (3.39–3.20)	3905 (647)	1607 (259)	30.1 (30.8)	3.77 (1.3)	96 (61)	29 (88)	0.44
3	30 to 62	24.5–3.2 (3.39–3.20)	6183 (962)	2290 (362)	42.9 (43.4)	1.69 (0.6)	86 (32)	84 (226)	0.67
4	30 to 50	24.5–3.2 (3.39–3.20)	3894 (631)	1568 (251)	29.6 (30.0)	2.64 (1.0)	89 (34)	49 (125)	0.46
5	10 to 30	28.5–3.2 (3.40–3.20)	3847 (629)	1524 (248)	29.2 (30.0)	3.27 (1.7)	93 (74)	32 (66)	0.41
6	50 to 70	22.7–3.2 (3.39–3.20)	3158 (500)	1288 (199)	24.1 (24.0)	1.14 (0.5)	74 (33)	83 (198)	0.45
7	−30 to 30	30.5–3.2 (3.39–3.20)	12088 (2062)	2140 (355)	40.1 (49.5)	3.10 (0.9)	95 (45)	55 (193)	0.52
Merged	—	29.5–3.2 (3.39–3.20)	39743 (6553)	4221 (680)	79.4 (81.3)	3.53 (1.5)	97 (61)	62 (175)	
Data reduction for crystal II, positioned parallel to the rotation axis (*a* = 30.70, *b* = 83.24, *c* = 117.34 Å)									
1	30 to 64	34.0–3.4 (3.61–3.40)	5562 (869)	2286 (350)	50.5 (51.6)	0.98 (0.4)	76 (23)	110 (307)	0.67
2	−68 to −50	28.0–3.2 (3.39–3.20)	3514 (543)	1623 (249)	30.2 (29.6)	0.90 (0.4)	55 (20)	126 (290)	0.39
3	30 to 62	28.0–3.2 (3.39–3.20)	6448 (1092)	2592 (439)	47.9 (51.6)	1.26 (0.4)	75 (20)	110 (300)	0.38
Merged	—	33.7–3.2 (3.39–3.20)	16613 (2737)	2973 (484)	55.2 (57.3)	1.24 (0.5)	83 (30)	130 (282)	
Merged data from both crystals (*a* = 30.59, *b* = 82.25, *c* = 117.76 Å)									
		33.7–3.2 (3.39–3.20)	56190 (9163)	4729 (744)	88.8 (89.7)	3.29 (1.4)	94 (53)	80 (220)	

## Data Availability

The atomic coordinates and structure factors for the Gab1–SHP2 complex structure have been deposited in the Protein Data Bank under accession code 9qcd. All other data supporting the findings of this study are available from the corresponding author upon reasonable request.
