# Supplementary material for: Strategies for mitigating radiation damage and improving data completeness in 3D electron diffraction of protein crystals
Source: Acta Crystallogr D Struct Biol. 2026 Jan 1;82(Pt 1):11–22. doi: 10.1107/S2059798325011258 (PMC12809435; doi:10.1107/S2059798325011258)
Supplement: Supplementary file 1 [file d-82-00011-sup1.pdf]

STRUCTURAL
BIOLOGY

Volume 82 (2026)

Supporting information for article:

Strategies for mitigating radiation damage and improving data completeness in 3D electron diffraction of protein crystals

Alaa Shaikhqasem, Farzad Hamdi, Lisa Machner, Christoph Parthier, Constanze Breithaupt, Fotis L. Kyrilis, Stephan M. Feller, Panagiotis L. Kastritis and Milton T. Stubbs

S1. Supplementary Methods: General microscope alignment and calibration

To capture electron diffraction data effectively, we tuned the microscope to achieve the lowest possible incident beam fluence, preventing pixel saturation and blooming. To accomplish this, both the gun lens and C1 lens were set to their maximum values in our microscope (i.e., #8 and #11, respectively). The specific settings used with the EPU-D software are summarized in Supplementary Table S1.

Table S1 Data collection parameters in EPU-D on the Glacios microscope, all using Gun Lens number 8 and Ceta-D camera

Preset	Optical Mode	Spot Number (C1)	Intensity (C2)	Magnification (Mag)<br>Camera Length (CL)	C2 Aperture	Defocus
Diffraction Acquisition	Nano-probe (Diffraction)	11	Parallel Beam Value	1.75 m (CL)	50 µm	-
Imaging Acquisition	Nano-probe (SA)	11	Parallel Beam Value	13500 X (Mag)	50 µm	-5 µm
Search<br>Auto-Eucentric	Microprobe (SA)	1	0.600<br>20 µm Beam Diameter	6700 X (Mag)	50 µm	-30 µm
Gridsquare	Microprobe (LM)	1	0.833	510 X	50 µm	-100 µm
Atlas	Microprobe (LM)	1	1.096	210 X	150 µm	-500 µm

To ensure minimal distortion in the diffraction pattern, particularly for higher resolution rings, and to maintain a consistent, well-defined camera length across all datasets, we aligned the microscope for parallel beam illumination. The procedure for achieving a parallel beam is detailed in the following sections, especially the section (d).

Enhancing the signal-to-noise ratio (SNR) requires confining the diffracting electron beam within the crystalline domain. A larger beam introduces unwanted scattering from the amorphous carbon or vitrified water background, degrading the diffraction quality. There are two primary methods to achieve beam confinement: (a) Selected Area Electron Diffraction (SAED), which utilizes an aperture in the image plane of the objective lens to select a specific region of interest, and (b) nanoprobe mode, where the pre-field of the twin lens compresses the beam into a well-defined, smaller probe. We opted for nanoprobe mode over SAED for several reasons:

(i) SAED does not restrict the irradiated area, but rather selects electrons originating from a specific region of the sample. Consequently, the beam damage area extends beyond the diffracting domain, making it challenging to apply low-dose techniques.

(ii) SAED lacks precision, as the electrons contributing to the final diffraction pattern are not strictly confined to the selected region (Williams & Carter, 2009).

Another critical parameter is the beam diameter. While large beams introduce the amorphous background noise, excessively small beams reduce the diffracting domain, leading to a lower signal-to-noise ratio (SNR) and, in extreme cases, broadening of the diffraction spots. To balance these factors, we selected a beam size of 1.7 µm, corresponding to a 50 µm C2 aperture in nanoprobe mode on the microscope used in this study. Given the dimensions of our microcrystals, this beam size maximized the diffracting domain while minimizing background contributions from the amorphous substrate.

We followed the precise alignment and calibration procedure in the following sequence, using a sample of nanometric particles of thallium chloride on a lacey carbon support film grid (Agar Scientific S110):

(a) The microscope and sample were set up in the "Eucentric Focus" condition to ensure that the region of interest (ROI) on the sample stayed in focus and at the correct Eucentric Height (EH) within the imaging preset. To achieve this, precise EH adjustments were made using the standard stage tilt method within a ±30° range. Subsequently, the microscope was configured to the imaging preset, except for a temporarily lower spot number for better visibility. Following that, the focus of the objective lens was manually adjusted on the sample, which had previously been positioned at the EH, and the current value of the objective lens was recorded as the calibrated Eucentric-Focus setting.

(b) The EPU-D software was calibrated for the minimum image shifts at various magnifications using the standard method provided by the software [EPU-D User Manual Section 5.2].

(c) For lower magnification presets (*i.e.* Search/Auto-Eucentric, Gridsquare and Atlas), we performed beam and C2 aperture centering to prevent any image cutoff.

(d) In the nano-probe mode, specifically for Imaging and Diffraction Acquisition (with the twin-lens pre-field active), we initially aligned the microscope to achieve symmetrical parallel beam illumination around the optical axis (Hamdi *et al.*, 2020). The steps for such general microscope alignment are summarized in Supplementary Figure S1. As it is challenging to see the edge of the objective aperture at very low beam intensities, (i.e. at spot number 11 and gun lens 8), the edge of the aperture was brought into focus at a temporarily lower spot number with increased beam current. While objective fine alignments like astigmatism,

current centering and coma-free alignment are not usually necessary for diffraction experiments, it is advisable to establish sound initial conditions, particularly for microscopes less commonly used for 3D-ED/MicroED.

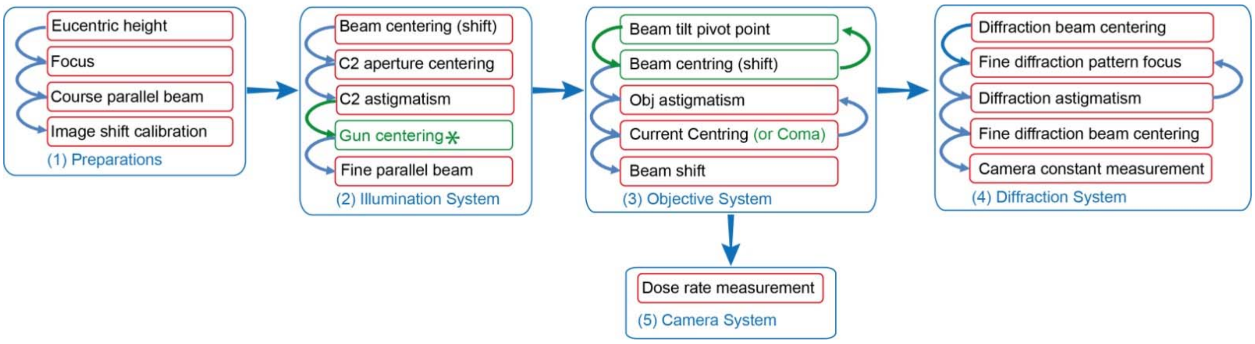

Figure S1 Flow chart of microscope adjustment and alignment before data acquisition. Usually, items in green should not have to be adjusted.

(e) We centered and fine-focused the diffraction pattern and adjusted the diffraction lens astigmatism. The microscope parameters in fully aligned and focused diffraction mode under nano-probe parallel beam conditions were then set as the diffraction acquisition preset in the EPU-D software. The values of the astigmators, deflectors, and optical state were recorded separately as an alignment file and gun register. Particular attention was paid to recording the values of diffraction and objective lenses, ensuring precise diffraction and image focus.

(f) Using the same thallium chloride sample, we calibrated the camera constant and subsequently the camera length based on the 200 kV accelerating voltage. For this purpose, we recorded several diffraction patterns and measured the positions of numerous Friedel pairs to determine the beam centre and respective 2θ angles. In this study, the camera length on the Ceta-D camera, at the nominal camera length of 1750 mm, was calculated to be 1704.5 mm, equivalent to a camera constant of $3.0544 x 10^3$ pixel.Å. Although it is possible to determine the camera length based on the final data, a high-fluence diffraction pattern from a hard material yields a more accurate measurement.

We measured the fluence at each preset meticulously to be able to track the dosage applied to each crystal. Accurate quantification of the fluence can be challenging, particularly when using the Ceta-D camera in diffraction mode or when the aperture or grid bars partially obstruct the beam. As our microscope lacks a Faraday cup, we employed the Falcon III EC direct electron detector in counting mode for precise fluence quantification. Ensuring that the microscope was in the previously aligned state, the sample was removed and the magnification adjusted manually, leaving all other parameters unchanged. Once the fluence fell within the "green zone" (indicating an acceptable range) in the Falcon III EC reference image manager software associated with the direct electron camera, a 10-

second exposure image in counting mode was captured and the average fluence from the resulting image was calculated.

Immediately prior to each data collection session, the camera gain reference was updated using the standard protocol with the CetaD reference image manager software to ensure the accuracy of our data acquisition process. The microscope was now ready for loading vitrified grids containing submicrometer protein crystals.

S2. Supplementary tables

Table S2 Data collection statistics for initial crystals of the Gab1-SHP2 complex (space group $P2_12_12_1$, $\alpha = \beta = \gamma = 90°$; all data collected at an accelerating voltage of 200 kV, corresponding to an electron wavelength of 0.025 Å).

Values given in parentheses are for the highest resolution shell.

acquisition	Angular range collected (°)	Resolution (Å)	Total reflections	Total unique reflections	Completeness (%)	I/σ (I)	$CC_{1/2}$ (%)	R-meas (%)	Mosaicity (°)
**Data reduction for crystal A** (a = 30.5 Å, b= 79.0 Å, c = 120.6 Å)									
1	-45 – -20	16–3.2 (3.50–3.2)	4766 (1149)	1554 (361)	29.5 (30.1)	3.49 (1.11)	96 (44)	35 (133)	0.38
2	-20 – 20	16–3.2 (3.50–3.2)	7845 (1906)	1645 (373)	31.2 (31.1)	1.31 (0.8)	40 (22)	83 (150)	0.44
3	20 – 45	16–3.2 (3.50–3.2)	4654 (1116)	2005 (474)	38.1 (40)	2.28 (0.59)	92(31)	48 (202)	0.55
merged	-	17–3.2 (3.50–3.2)	17256 (4173)	3558 (815)	67.7 (68)	2.41 (0.92)	53 (31)	75 (161)	
**Data reduction for crystal B** (a = 30.60 Å, b= 80.07 Å, c = 120.35 Å)									
1	-45 – -20	17–3.2 (3.50–3.2)	4657 (1124)	1734 (404)	33 (33.7)	2.3 (0.53)	96 (9)	48 (275)	0.52
2	-20 – 20	17–3.2 (3.50–3.2)	7977 (1906)	1695 (384)	32 (32)	1.3 (0.94)	23 (10)	91 (116)	0.45
3	20 – 45	17–3.2 (3.50–3.2)	4850 (1163)	1797 (418)	34 (35)	1.13 (0.84)	21 (20)	48 (202)	0.31
merged	-	17–3.2 (3.50–3.2)	17492 (4190)	3213 (734)	61 (61.3)	1.5 (0.86)	32 (14)	93 (134)	
**Merged data from both crystals** (a = 30.55 Å, b= 79.50 Å, c = 120.48 Å)									
		17 – 3.2 (3. 35 –3.2)	34748 (8363)	3631 (831)	69 (69.4)	2.57 (1.12)	58 (24)	92 (147)	

Table S3 Refinement statistics for Gab1-SHP2 complex structure.

Values given in parentheses are for the highest resolution shell.

**pdb code**	9qcd
Resolution range (Å)	33.72-3.20
Completeness (%)	88.8 (89.7)
No. of reflections, working set	4487
No. of reflections, test set	238
Final R_{cryst}	30.1
Final R_{free}	35.3
**No. of non-H atoms**	
Protein	1642
Peptide	290
Ions	0
Ligands	0
Waters	0
Total	1932
**R.m.s. deviations from ideality**	
Bonds (Å)	0.003
Angles (°)	0.648
**Average *B* factors (Å^2)**	
Protein	48.96
Peptide	65.95
Ions	-
Ligands	-
Waters	-
**Ramachandran plot**	
Favoured (%)	95.6
Allowed (%)	4.4
Outlier (%)	0.0

S3. Supplementary figures

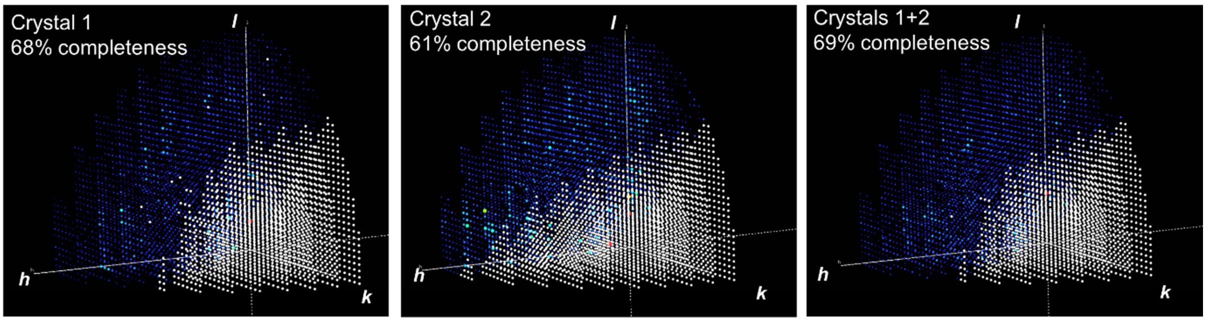

(*a*) (*b*) (*c*)

Figure S2 Completeness of data collected from initial GAB1-SHP2 crystals. 3D reciprocal space representation of data collected from the first crystal in (*a*). Corresponding data from a second crystal (*b*) were 61% complete but represented the same region of reciprocal space, indicating a similar orientation on the grid; the merged data from both crystals showed no increase in reciprocal space coverage (*c*). Missing reflections are depicted in white. 3D representation of reflection spheres was prepared using 3D Data Viewer in Phenix (Adams *et al.*, 2010).

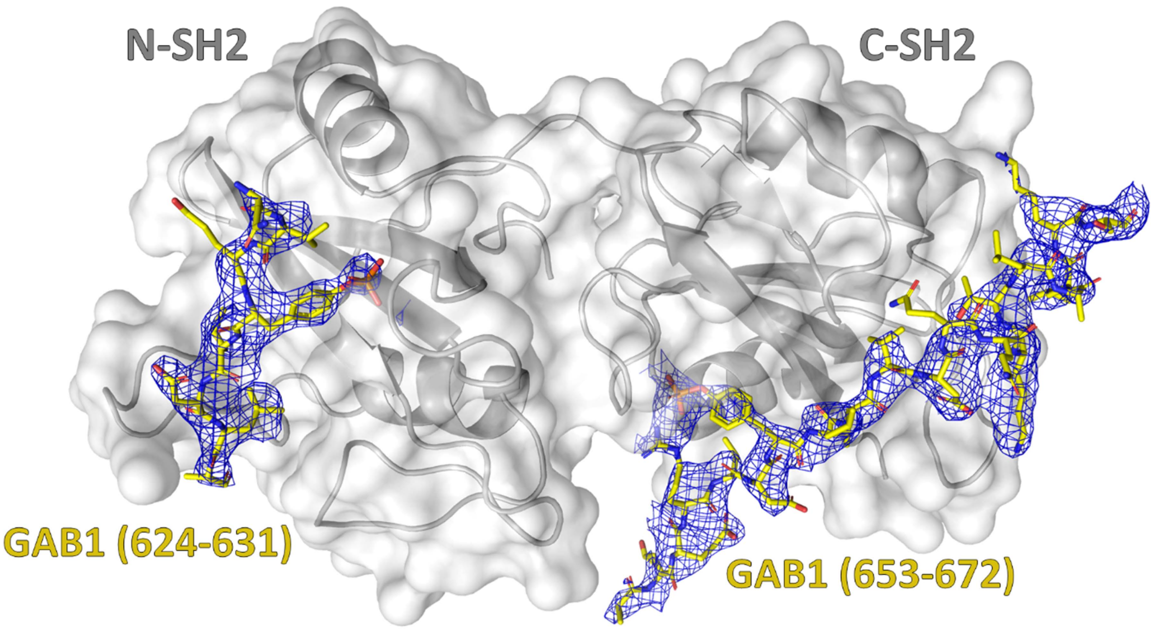

Figure S3 Crystal structure of the Gab1$^{617\text{-}684}$ fragment in complex with SHP2$^{1\text{-}222}$ (Machner *et al.*, 2025) determined by 3D-ED/MicroED. SHP2 is depicted in cartoon representation (dark grey) with a light grey surface; ordered parts of the Gab1 peptide are shown as yellow sticks with corresponding $2F_o$-F_c density (contoured at 1.5 σ, blue mesh).

References

Adams, P. D., Afonine, P. V., Bunkóczi, G., Chen, V. B., Davis, I. W., Echols, N., Headd, J. J., Hung, L. W., Kapral, G. J., Grosse-Kunstleve, R. W., McCoy, A. J., Moriarty, N. W., Oeffner, R., Read, R. J., Richardson, D. C., Richardson, J. S., Terwilliger, T. C. & Zwart, P. H. (2010). *Acta Crystallogr D Biol Crystallogr* **66**, 213–221.

EPU-D User Manual: https://documents.thermofisher.com/TFS-Assets/MSD/Product-Guides/epu-d-user-manual-1-22.pdf

Hamdi, F., Tüting, C., Semchonok, D. A., Visscher, K. M., Kyrilis, F. L., Meister, A., Skalidis, I., Schmidt, L., Parthier, C., Stubbs, M. T. & Kastritis, P. L. (2020). *PLoS One* **15**.

Machner, L., Shaikhqasem, A., Gruber, T., Hamdi, F., Breithaupt, C., Kniest, J., Wiebe, F., Lewitzky, M., Parthier, C., Kyrilis, F. L., Balbach, J., Kastritis, P. L., Feller, S. M. & Stubbs, M. T. (2025). *Structure*, in press.

Williams, D. B. & Carter, C. B. (2009). Transmission electron microscopy: A textbook for materials science Springer US.
